# Medical leaders or masters?—A systematic review of medical leadership in hospital settings

**DOI:** 10.1371/journal.pone.0184522

**Published:** 2017-09-14

**Authors:** Mathilde A. Berghout, Isabelle N. Fabbricotti, Martina Buljac-Samardžić, Carina G. J. M. Hilders

**Affiliations:** Erasmus School of Health Policy & Management, Erasmus University Rotterdam, Rotterdam, The Netherlands; TNO, NETHERLANDS

## Abstract

Medical leadership is increasingly considered as crucial for improving the quality of care and the sustainability of healthcare. However, conceptual clarity is lacking in the literature and in practice. Therefore, a systematic review of the scientific literature was conducted to reveal the different conceptualizations of medical leadership in terms of definitions, roles and activities, and personal–and context-specific features. Eight databases were systematically searched for eligible studies, including empirical studies published in peer-reviewed journals that included physicians carrying out a manager or leadership role in a hospital setting. Finally, 34 articles were included and their findings were synthesized and analyzed narratively. Medical leadership is conceptualized in literature either as physicians with formal managerial roles or physicians who act as informal ‘leaders’ in daily practices. In both forms, medical leaders must carry out general management and leadership activities and acts to balance between management and medicine, because these physicians must accomplish both organizational and medical staff objectives. To perform effectively, credibility among medical peers appeared to be the most important factor, followed by a scattered list of fields of knowledge, skills and attitudes. Competing logics, role ambiguity and a lack of time and support were perceived as barriers. However, the extent to which physicians must master all elicited features, remains ambiguous. Furthermore, the extent to which medical leadership entails a shift or a reallocation of tasks that are at the core of medical professional work remains unclear. Future studies should implement stronger research designs in which more theory is used to study the effect of medical leadership on professional work, medical staff governance, and subsequently, the quality and efficiency of care.

## Introduction

Recently, medical leadership in hospitals has received increasing attention from both scholars and practitioners. Medical leadership is considered to play an important role in improving organizational performance, including the quality of care, patient safety and cost-efficient care [1, 2, 3, 4]. Furthermore, many argue that medical leadership is necessary for overcoming the divide between medical and managerial logics in hospitals that hampers improvement in healthcare [5, 6]. However, despite the popularity of this topic, the scientific conceptualization of medical leadership remains ambiguous [7].

One stream of scientists conceptualizes medical leadership as formal management roles played by physicians. These authors refer to the administrative roles of physicians [6, 8, 9, 10] by using the term medical leadership interchangeably with the term medical management. This conceptualization stems from the historical introduction of a medical manager to hospitals in many countries (USA [11], UK [12], Australia [13], and the Netherlands [6]) as a response to difficulties in hospital governance to ‘control’ and ‘manage’ medical professionals [14, 15].

Traditionally, professional and managerial logics are portrayed as intrinsically conflicting [16, 17]. On the one hand many scholars argue that physicians are ‘infringed’ by managerial logics following the rise of New Public Management [18] in the public sector. Due to an increase in managerialism in healthcare, professional work is increasingly standardized, regulated and specified in terms of quality indicators, which supposedly led to a decrease in professional autonomy and work satisfaction [3]. On the other hand, professionals are often portrayed as resistant to organizational and governmental requirements [19] and therefore are difficult to control.

To overcome the assumed divide between professional and managerial logics, hospitals have introduced the role of the medical manager, who ought to perform as a so-called ‘linking pin’ [6] between management and professionals. This was based on the idea that physicians are more influenced by their peers than by managers, due to the highly socialized character of the medical profession [6, 15, 16]. A well-known example of this strategy is the introduction of clinical directorates, which was first achieved in the USA at John Hopkins Hospital in Baltimore, followed by the UK at Guy’s Hospital, in which a clinical director was responsible for, among other things, quality and the budget in her/his directorate.

Another stream of literature represents medical leadership as an intrinsic component of physicians’ daily work [5, 20, 21]. That is, physicians must act as ‘leaders’ within their clinical role, by organizing clinical work and establishing cross-departmental collaboration, thereby aiming for high-quality and cost-efficient care. As such, this leadership role is an informal role that transcends formal managerial work and thus applies to *all* physicians [1, 4, 5, 20, 21]. Subsequently, a call for training medical doctors in managerial and leadership skills arose [1, 22, 23, 24]. To prepare physicians for their leadership roles, existing competency models such as the well-known CanMEDS model [25] added leadership skills to the medical and technical skills. Furthermore, new competency models have evolved, such as the Medical Leadership Competency Framework in the UK [26] and the Framework Medical Leadership in the Netherlands [27] that particularly focus on managerial and leadership skills.

Although a distinction can be made between the literature streams, a clear demarcation of the concept of medical leadership remains absent. Therefore, a better understanding of the concept is necessary for both research and practice. First, the lack of a conceptual understanding and commonly used terminology hampers empirical developments in research. Second, the lack of a clear conceptualization appears to be problematic for physicians in performing their medical leadership roles in practice; their roles are poorly understood [28, 29], and physicians encounter identity struggles [8, 29], experience stress [30] and a lack of time [31] and feel unsupported [12] and unprepared [32]. If medical leadership is important for improving the quality of care, cost efficiency and hospital governance, it is necessary to first obtain a better understanding of the nature of medical leadership, the activities and roles performed by medical leaders carry, the skills that are necessary and the influential factors. Therefore, the aim of this systematic review is to unravel the different conceptualizations of medical leadership. In this systematic review, we aim to provide an overview of the scientific literature regarding the definitions of medical leadership, the activities and roles performed by a medical leader, the required knowledge and skills, and the influential factors.

## Methods

### Search strategy

The following eight databases were systematically searched for eligible studies: Embase, Medline, Web of Science, PubMed, Cochrane, CINAHL, ABI/inform and Google Scholar. The search strategy was established in collaboration with a librarian from a medical library who is a specialist in designing systematic reviews. The search included terms related to physicians, management and leadership, skills and influential factors. A preliminary exploratory literature search of our topic illustrated the diversity in the terms used to describe physicians in leadership or managerial roles. Therefore, we adopted a broad search strategy, which yielded a large number of articles. The following search terms were used: medical, clinical, physician*, clinician*, doctor* AND (combined with) lead*, manage*, executive*, director*, ceo* (see S1 Appendix for an example of the full electronic search strategy for all databases). The final search was performed on January 31, 2017.

### Eligibility criteria

Studies were included if they met the following inclusion criteria:

- Type of participants–Medical leaders who were defined in this study as physicians in a management or leadership role who work in a hospital setting.
- Topic–Studies should have focused on (1) definitions of medical leadership, (2) activities and roles performed by medical leaders, (3) skills supposedly required for a medical leader, or (4) influential factors that were experienced as barriers or facilitators in performing a medical leadership role.
- Type of publication–Empirical studies published in peer-reviewed journals are eligible. Medical leadership is a popular topic in the gray literature, however, an overview of empirically based knowledge regarding medical leadership is lacking. Therefore, we aimed to describe the empirical knowledge of medical leadership and decided to not include the gray literature. Empirical studies could include all research designs, except for systematic reviews.
- Language–Studies should be written in English.

We did not make any restrictions for the year of publication.

### Record selection

**Fig 1:**
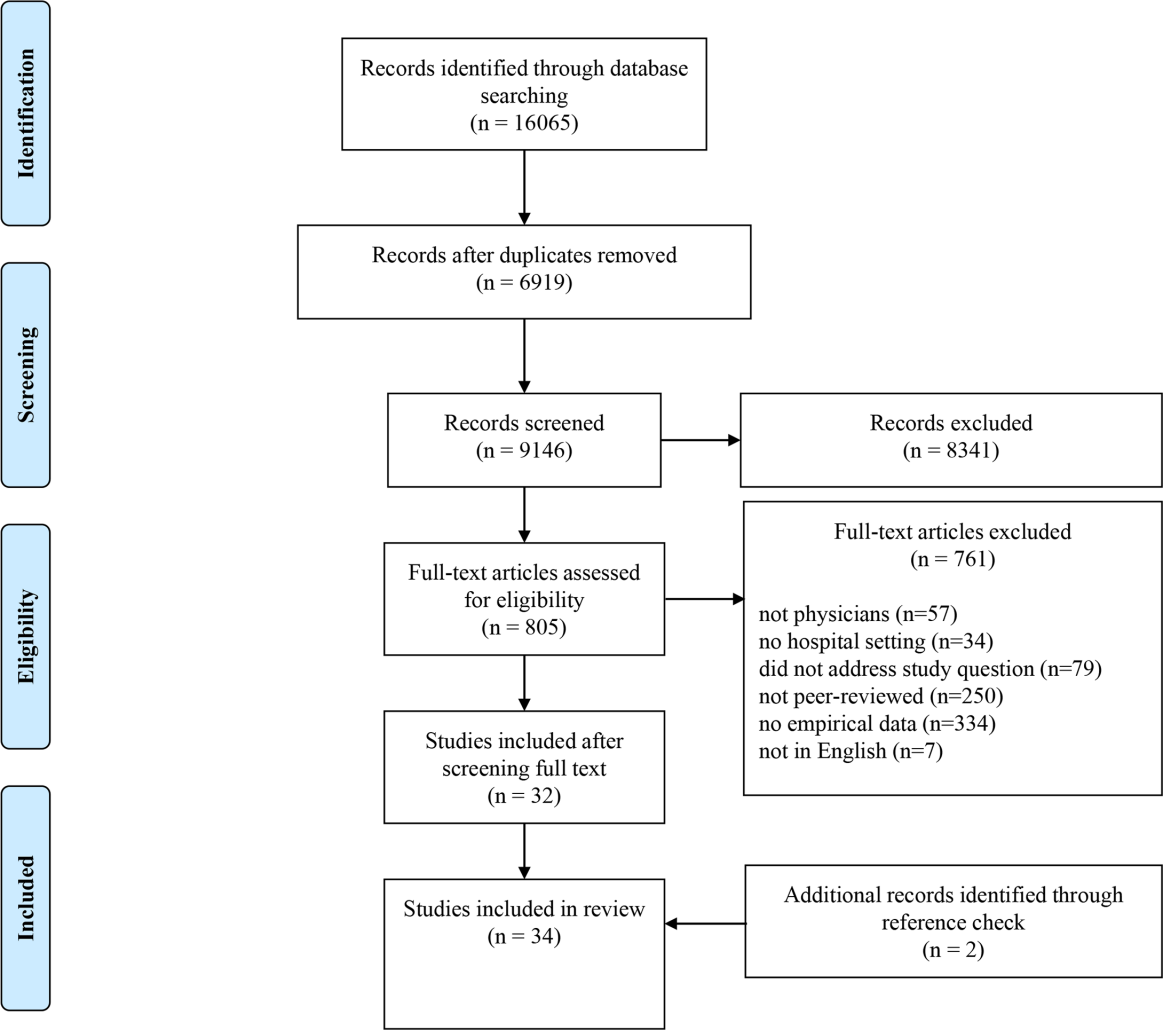
Flow diagram of the record selection.

The search yielded 16,065 articles. After excluding the duplicate studies, 9,146 articles remained for screening (Fig 1). The screening process consisted of three steps. First, two researchers (MB and IF) independently screened all articles by scanning the titles and abstracts. Articles were excluded if they did not meet all inclusion criteria. If the information provided in either the title and/or the abstract was insufficient for a justified decision, the articles were included in the full-text screening phase. Although this resulted in a large number of full-text articles to review, it allowed us to be as thorough as possible during this phase. Second, 805 full-text articles were examined for eligibility. The first reviewer (MB) performed the first screening of the full texts for inclusion and excluded all articles that obviously did not meet the inclusion criteria. Both reviewers (MB and IF) independently screened the remaining articles by closely reading the full texts. Finally, a reference check of the included articles was performed, resulting in the inclusion of two additional articles.

### Data analysis

**Fig 2:**
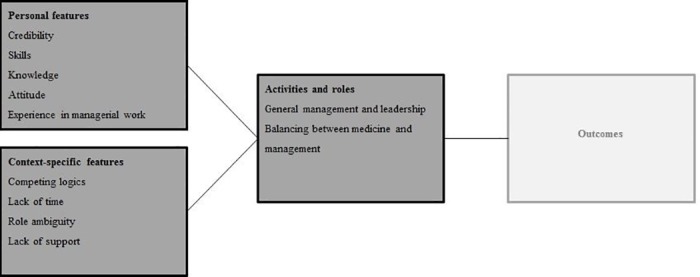
Conceptual framework of medical leadership in hospital settings.

To analyze the data, we conducted a narrative analysis of the review material. Due to the heterogeneity in the included records in terms of the conceptualizations of medical leadership, study aims and research designs, a meta-analysis was neither suitable nor possible. According to previous recommendations [33] for a narrative analysis in systematic reviews, we conducted the following steps: first, we created a data extraction form for each study, in which we summarized the author(s), year of publication, journal, country, methods used, definitions, activities and roles, skills, and influential factors. The data regarding the activities and skills yielded some overlap, for example, ‘influencing’ was considered a skill by some, while other authors described ‘influencing’ as an activity. To ensure objectivity, we relied on the terms used by the author(s) while summarizing the data into the extraction form. Then, we inductively coded the data in three overarching themes. *The first theme* represented items that referred to similar activities and roles. We clustered these items into the following two broad categories: (1) general management and leadership and (2) balancing between management and medicine. *The second theme* represented items that referred to skills, which were clustered into the following three categories: (1) knowledge, which was defined as the understanding of a certain subject; (2) skills, which were defined as the ability to accomplish something; and (3) attitude, which was defined as a personal characteristic. We determined the category to which each item fitted best. *The third theme* represented the data regarding influential factors. We clustered the items that referred to the same factor into the following six categories: (1) credibility, (2) experience in management, (3) competing logics, (4) role ambiguity, (5) lack of support, and (6) lack of time. After all themes were identified, we distinguished the personal from the context-specific features. These features can be either barriers or facilitators, depending on the specific person or context it relates to. At the personal level, these features refer to characteristics that are associated with a medical leader. At the context-specific level, these features refer to the cultural and institutional characteristics of the hospital in which a medical leader works. Finally, the personal features, context-specific features, and activities and roles were mapped into a comprehensive conceptual framework that will guide the results (Fig 2).

## Results

### Research methods, journals and countries

Most studies had a qualitative research design (N = 24). Of these qualitative studies, some studies relied on interviews (N = 11), while the other studies used a combination of interviews, observations, document analyses or focus groups (Table 1). The remaining studies had a quantitative research approach (N = 7) using self-administered surveys in which the participants listed and/or ranked task inventories or skills, or a mixed methods study design (N = 3) in which the researchers used a combination of interviews and surveys. All survey studies were cross-sectional and thus contained no longitudinal data.

**Table 1:** Details of the studies included in this review (n = 34).

Authors (year of publication)	Study aim	Methodology, participants, and setting	Country
Andersson 2015 [8]	To analyze the identity challenges that physicians with medical leadership positions face	Interviews and observations. Participants: physicians (N = 20) including physicians with a managerial role (N = 10), managers (N = 8) and their peers and subordinates (N = 24). Observations (N = 11) occurred during meetings involving physicians and managers. Setting: four hospitals	Sweden
Barrable 1988 [34]	To explore the role of the physician manager to outline administrative performance	Surveys and interviews. Participants: physician managers (N = 13) completed the survey. Interviews were held with physician managers (N = 16), the chairman (N = 1) and the president of the medical staff (N = 1) Setting: academic hospital	Canada
Betson & Pedroja 1989 [11]	To describe the job of physician managers in hospitals	Survey containing a task inventory. Tasks were rank-ordered according to the frequency and responsibility of the task. Participants: medical directors (N = 502). Setting: hospital (N = unknown)	USA
Buchanan et al. 1997 [12]	To explore how doctors engage in hospital management processes and consider the implications of current experiences in the next generation of clinical directors	Interviews. Participants: clinical directors (N = 6) and other hospital management team members, the chief executive, non-clinical directors, business managers and senior nurse managers (N = 19). Setting: general teaching hospital	UK
Dawson et al. 1995 [35]	To examine the role of clinical directors and their increasing involvement in management and competition	Interviews and a survey. Participants: clinical directors (N = 50), medical directors (N = 9), executive directors (N = 40), senior executives (N = 45) and clinical directors who participated in a management development program (N = 15). Setting: NHS trusts (N = 21)	UK
Dedman et al. 2011 [13]	To explore the perceptions of clinical directors and their roles and needed skills in clinical directorates	Interviews and document analysis. Participants: clinical directors (N = 13), chief executives (N = 3), nursing directors (N = 12), business managers (N = 9), and department heads (N = 2). Setting: public teaching hospitals (N = 3)	Australia
Dine et al. 2010 [36]	To discover the characteristics associated with effective physician leadership	Focus groups. Participants: physicians (N = 6), interns (N = 6) residents (N = 7) and nurses (N = 5). Setting: academic hospital	USA
Dwyer 2010 [28]	To document the roles, perceived skills, attributes and experience required of medical administrators	Interviews. Participants: directors of medical services (N = 14). Setting: eight metropolitan public hospitals	Australia
Hallier & Forbes 2005 [37]	To understand how organizational professionals perceive the introduction of managerialism and the incorporation of managing into specialist roles	Interviews. Participants: clinical directors (N = 18). Setting: NHS acute/district general hospitals (N = unknown)	Scotland
Holmboe et al. 2003 [38]	To investigate the characteristics and skills of physicians involved in improving quality	Interviews. Participants: key physicians, nurses, and quality management and administrative staff (N = 45). Setting: eight hospitals	USA
Hopkins et al. 2015 [39]	To determine the particular competencies demonstrated by effective physician leaders	Interviews. Participants: physicians who participated in a leadership development program (N = 28). Setting: academic hospital	USA
Kindig & Lastirir-Quiros 1989 [40]	To understand the nature of the administrative roles currently performed by physician executives and their perceptions of changes in these roles in the future	Survey. A task inventory was used to rank 33 tasks according to importance. Participants: physician executives (N = 159). Setting: different hospitals	USA
Kippist & Fitzgerald 2009 [31]	To examine the tensions between hybrid clinical managers' professional values and health care organizations’ management objectives	Interviews and observations. Participants: physician-managers who participate in a clinical leadership development program (N = 7), their staff (N = 3), the clinical leadership development facilitator (N = 1) and senior managers (N = 3). Observations of interactions between team members at several team meetings. Setting: large teaching hospital	Australia
Kuhlmann et al. 2016 [41]	To explore the gaps and organizational weaknesses that may constrain new forms of more integrated (or hybrid) clinical management	Interviews. “Participants: physicians without a management position (N = 6) and physicians with a management position (N = 6) Setting: four departments at one urban hospital and three different hospitals	Sweden
Leigh & Newman 1997 [42]	To describe the tasks of medical directors and the problems associated with their new role	Survey. Participants: medical managers (N = 236) including 14 mini case-studies of current job holders and a broad outline of the responsibilities of medical managers. Setting: hospital (N = unknown)	UK
Llewellyn 2001 [43]	To understand the aspirations and activities of clinical directors	Interviews. Participants: clinical directors (N = 16). Setting: three hospitals	UK
Meier 2015 [2]	To explore how leadership is practiced across four different hospital units	Interviews, observations and document analysis. Participants: physicians (N = 5), nurses (N = 4), and a physiotherapist (N = 1). Setting: four hospital units, in two different hospitals	Denmark
Mo 2008 [44]	To determine the role of physician-managers after unitary management reforms	Interviews. Participants: medical managers (N = 10). Setting: university hospital	Norway
Ong 1998 [32]	To examine the way in which clinicians and their clinical teams are developing their understanding of the new role	Interviews. Participants: clinical directors (N = 2), their managing pairs (N = unknown) and the executive team (N = unknown). Setting: two directorates in a general hospital	UK
Palmer at al. 2009 [45]	To explore the perceptions of junior doctors of the most important leadership competencies	Survey. One on competencies and one on leadership styles (ranking). Participants: year-2 physicians (N = 196). Setting: nine hospitals	UK
Quinn & Perelli 2016 [46]	To understand how physician leaders construe their roles	Interviews. Participants: full-time physician administrators (N = 6), physicians who are either department chairs or presidents of staff (N = 12) and physician leaders without a formal leadership role (N = 6). Setting: four hospitals	USA
Pepermans et al. 2001 [47]	To determine the job tasks of medical directors and head nurses in intensive care units	Interviews, observations and focus groups. Participants: medical directors (N = unknown), observational units of activities (N = 235), focus groups (N = unknown) and medical directors and head nurses (N = 3–6) Setting: six hospital IC units	Belgium, Denmark, Portugal, Switzerland, Netherlands
Robinson et al. 2013 [48]	To determine the personal and professional characteristics of medical leaders in urology compared to other specialties	Survey (listing of duties and skills). Participants: program directors and department heads of urology (N = 13) and other specialties (N = 88). Setting: hospital (N = unknown)	Canada
Rotar et al. 2016 [49]	To explore the formal managerial roles of doctor managers in hospitals and to determine the association between the level of their involvement in hospital management and the level of implementation of quality management systems	Survey and interviews. Participants: (1) country experts (N = 19) in the OECD's health care quality indicator program and (2) physicians that have a formal or informal leading role (N = 1,670). Setting: 188 hospitals	Europe, Israel, Japan, Singapore, South Korea, Turkey
Spehar et al. 2015 [50]	To investigate how clinicians' professional background influences their transition into the managerial role and identity as clinical managers	Interviews and observations. Participants: physicians (N = 13), nurses (N = 16) and a clinician with another healthcare background (N = 1). Setting: four public hospitals	Norway
Spyridonidis et al. 2015 [51]	To understand how physicians assume a 'hybrid' role and identity processes as they take on managerial responsibilities	Interviews pre -and post, observations and document analysis. Participants: physician managers (N = 62), pre -and post project (total N = 124 interviews), and CLAHRC senior members (total N = 210 interviews). Setting: hospital (N = unknown)	UK
Taylor et al. 2008 [52]	To explore the required leadership qualities, knowledge and skills among medical leaders in an academic hospital setting	Interviews. Participants: physicians who followed a leadership program (N = 10) and course and clerkship directors, program directors and department chairs (N = 8), and division directors and academic deans (N = 7). Setting: academic hospital	USA
Thorne 1997a [53]	To discover how clinicians became clinical directors, how they perceived and enacted their role and its impact on themselves and others	Interviews and observations. Participants: clinical directors (N = unknown). Observations at management board meetings and 'being around' in both formal and informal settings. Setting: Large provincial teaching hospitals trust	UK
Thorne 1997b [54]	To identify the perspectives of doctors in management and managers of the clinical director role	Interviews and observations. Participants: clinical directors (N = 14). Setting: 14 directorates within one NHS trust	UK
Vinot 2014 [9]	To explore the managerial roles of doctors after major hospital management reforms	Interviews and document analysis. Participants: At each hospital two interviews were held: one with a hospital director and one with a medical manager (total N = 10). Setting: three public and two university hospitals	France
Willcocks 1995 [30]	To suggest a possible framework for examining the effectiveness of individual directors	Interviews and document analysis. Participants: clinical directors and managers (N = unknown). Setting: NHS trust hospital	UK
Williams 2001 [10]	To indentify the skills and knowledge required for effective medical leadership	Survey containing a list of skills and knowledge, which was rank-ordered. Participants: physicians in executive or senior management positions (N = 111). Setting: hospital (N = unknown)	USA
Williams & Ewell 1996 [55]	To assess hospital medical staff governance and leadership characteristics	Survey (3 types). Participants: Two surveys were completed by the medical staff specialists, office managers or coordinators, and one by the chiefs of staff. Setting: 65 hospitals	USA
Witman et al. 2010 [6]	To obtain insights regarding the day-to-day practices of medical leaders	Interviews, observations, focus groups in small learning groups (N = 26, in 33 groups). Participants: department heads (N = 6), their colleagues, residents and non-medical managers (N = 23). Setting: three departments in a university hospital	NL

These studies were published in a wide array of journals ranging from purely clinical to exclusively management journals. Most studies were published in health management-related journals (N = 12), followed by studies in health (care)-related journals (N = 11), including leadership- and educational-associated journals. The remaining studies were published in management journals (N = 6), specific medical specialty journals (N = 2), an organizational journal (N = 1), a public sector journal (N = 1) and a human resource journal (N = 1).

Most studies were conducted in the UK (N = 11), followed by the USA (N = 9), Australia (N = 3) and Canada (N = 2) (Table 1). One study was performed in different countries in North America, Europe and Asia. The remaining studies were conducted in various other Western-European countries (N = 8).

### Definitions

Most studies did not explicitly define the concept of medical leadership. Implicitly, these leaders were described as “champions” [38] “key physicians”, “team-oriented” [48], “change agents” and “visionaries” [39]. These physicians are, “able to enact to multiple functions in addition to their clinical roles” [ibid], “committed to hospital success” [38] and able to “influence and inspire their colleagues” [6, 38, 39]. Only two studies provided an explicit definition of medical leadership, describing it as “embodied by a practitioner who operates as an opinion-leader or even as a particular school of thought within medicine” [9] and “physicians in leading positions” [8]. Although many researchers did not define medical leadership, they *did* underscore the need for a clear definition [36, 39].

In contrast, studies reporting formal medical leadership roles, which were explicitly defined as medical management, were more straightforward regarding the definition of medical management: “(senior) doctors who have assumed management responsibilities” [43] “who may or may not retain a role in clinical work” [50].

### Types of medical leadership

Despite the lack of a common definition, as indicated in the introduction, two types of medical leadership could be identified in the literature. Type 1 includes physicians in formal managerial roles and is, described in studies as either ‘medical management’ or ‘medical leadership’. The participants included in these studies were, medical directors working at the executive level [42] or clinical directors working at the management level [35]. The nature of the medical management function differed. In some studies, these positions were full-time affiliations in which the physicians ceased to perform clinical work [34], whereas in other cases, the positions were considered as a part-time jobs ‘on the side’, meaning that they will first and foremost be a physician [6], and finally, there were studies in which both full- and part-time variants were identified [31, 41].

Type 2 includes physicians in informal roles and is described in studies as ‘medical leadership’. The included participants in these particular studies were described as physicians who act as a leader within their daily clinical work, such as physicians who were involved in quality improvement projects [38].

### Activities and roles

Twenty-nine studies reported the activities and roles performed by medical leaders or assumed necessary for effectively performing such a role (Table 2). The resultant list included activities and roles capturing a broad range of topics. Two different types of activities and roles appeared. These two types include general management and leadership activities and balancing between management and medicine. We observed no distinction between activities specifically adhering to either formal or informal roles of medical leadership.

**Table 2:** Activities and roles.

**Authors (year of publication)**	**General management and leadership work**	Balancing between management and medicine*
Andersson (2015) [8]	-	Influencing for multiple objectives
Barrable (1988) [34]	Strategy, business planning, responsible for performance, finance, HR, decision making, policy, meetings	Influencing for multiple objectives
Betson & Pedroja (1989) [11]	Staff management, consensus building, communication, strategy, responsible for performance, finance, HR, decision making, committees, research and teaching, meetings, policy, negotiation	Bridging management and medicine, dealing with tensions, representing medical staff
Buchanan et al. (1997) [12]	Multidisciplinary collaboration, communication, staff management, responsible for performance, finance, HR, problem solving, administration, meetings	Influencing for multiple objectives, representing medical staff
Dawson et al. (1995) [35]	Multidisciplinary collaboration, staff management, leading a team, communication, strategy, business planning, responsible for performance, leading change, finance, negotiation, contracting, HR, networking	Bridging management and medicine, representing medical staff
Dedman et al. (2011) [13]	-	-
Dine et al. (2010) [36]	Strategy, finance, decision making, coordination and delegation, consensus building, administration, meetings, communication, policy, feedback, empowering others	-
Dwyer (2010) [28]	Multidisciplinary collaboration, staff management, strategy, responsible for performance, leading change, finance, clinical issues, HR, networking, research and teaching, legal issues, policy	Bridging management and medicine, influencing for multiple objectives
Hallier & Forbes (2005) [37]	Responsible for performance, finance	-
Holmboe et al. (2003) [38]	Committees, empowering others, multidisciplinary collaboration, consensus building, communication, feedback, responsible for performance, leading change	-
Hopkins et al. (2015) [39]	-	-
**Authors (year of publication)**	**General management and leadership work**	**Balancing between management and medicine**
Kindig & Lastirir-Quiros (1989) [40]	Multidisciplinary collaboration, staff management, consensus building, communication, strategy, business planning, policy, responsible for performance, leading change, finance, clinical issues, negotiation, HR, research and teaching, legal issues, networking, risk management, representing interests	-
Kippist & Fitzgerald (2009) [31]	-	-
Kuhlman et al. 2016 [41]	Administration, responsible for performance, staff management	-
Leigh & Newman (1997) [42]	Finance, contracting, strategy, networking, negotiation, responsible for performance, staff management, influencing, leading change, clinical issues, HR	Decision making, influencing for multiple objectives, bridging management and medicine
Llewellyn (2001) [43]	Finance, consensus building, responsible for performance, risk management, negotiation	Bridging management and medicine, influencing for multiple objectives, decision making
Meier (2015) [2]	Multidisciplinary collaboration, coordination and delegation	Negotiation, decision making
Mo (2008) [44]	Staff management, strategy, responsible for performance, leading change, HR, administration	Bridging management and medicine, role making
Ong (1998) [32]	Staff management, leading a team, strategy, networking, business planning	Role making, bridging management and medicine
Palmer et al. (2009) [45]	-	-
Pepermans et al. (2001) [47]	Staff management, consensus building, communication, responsible for performance, coordination and delegation, problem solving, networking, administration, meetings, decision making, empowering others	-
Quinn & Perelli 2016 [46]	Administration, meetings, HR, consensus building	Bridging management and medicine, influencing for multiple objectives
Robinson et al. (2013) [48]	Advising, finance, HR	-
**Authors (year of publication)**	**General management and leadership work**	**Balancing between management and medicine**
Rotar et al. 2016 [49]	Advising, HR, teaching, clinical issues, staff management, decision-making, finance	-
Spehar et al. (2015) [50]	Finance, administration, advising, empowering others	Influencing for multiple objectives, role making
Spyridonidis et al. (2015) [51]	Multidisciplinary collaboration, responsible for performance, leading change, research and teaching	Role making, coordination and delegation, negotiation, influencing for multiple objectives, bridging management and medicine
Taylor et al. (2008) [52]	-	-
Thorne (1997a) [53]	Staff management, strategy, responsible for performance, leading change, finance, contracting, meetings, negotiation	Influencing for multiple objectives, bridging management and medicine, role making, dealing with tensions
Thorne (1997b) [54]	Leadership by example, staff management, strategy, leading change, clinical issues, finance, contracting, networking	Decision making, influencing for multiple objectives
Vinot (2014) [9]	Multidisciplinary collaboration, staff management, strategy, responsible for performance, finance, coordination and delegation, contracting, HR, administration	Bridging management and medicine
Willcocks (1995) [30]	Leading a team, strategy, problem solving, decision making, negotiation,	Role making, representing medical staff
Williams (2001) [10]	Contracting, risk management, staff management, administration, strategy, finance, responsible for performance	-
Williams & Ewell (1996) [55]	Strategy, finance, committees	Representing medical staff, decision making
Witman et al. (2010) [6]	Staff management, feedback, advising, responsible for performance, influencing, leading by example, consensus building, meetings, communication	Bridging management and medicine, influencing for multiple objectives

* Features indicated with an asterisk indicate the unique features of medical leadership in contrast to those of general leadership

#### General management and leadership

Twenty-eight studies described 30 different activities. These activities were described as straightforward management or leadership duties, including finance (N = 18), strategy (N = 15), staff management (N = 17), human resources (N = 12), leading change (N = 9), or administration (N = 9) (Table 2). It is argued that these activities are performed—or should be performed—to achieve organizational and patient objectives, even when these activities conflict with personal or department goals [52], thereby stressing that medical leadership is a rather rational profession [56], in which medical leaders assume more responsibility for departmental performance (N = 17) in terms of outcomes (e.g., quality of care and costs) and of the functioning of individuals (e.g., medical colleagues) than ‘normal’ physicians. Furthermore, medical leaders should be more concerned with innovation and improvements in clinical issues (N = 5) and increasing multidisciplinary collaboration to improve the quality of care (N = 8). To achieve these objectives, medical leaders should, among other functions, influence (N = 2) and empower peers (n = 4), communicate information to medical peers as well as back and forth to management and medical practitioners (N = 8), build a consensus (N = 8) and resolve problems (N = 3). Moreover, medical leaders should lead or attend meetings (N = 8), network (N = 7) within and outside the hospital, negotiate (N = 6), contract (N = 6) and make decisions (N = 6). Additional activities that were mentioned more than twice include policy activities (N = 5), research and teaching (N = 5), business planning (N = 4), coordination and delegation (N = 4), advising (N = 4), committees (N = 3), leading a team (N = 3), providing feedback (N = 3), and risk management (N = 3).

#### Balancing between management and medicine

Other activities and roles appeared to be more specific to the context in which the medical leaders performed their roles: namely, on the border of management and medicine (represented by 20 studies, see Table 2).

#### Bridging the managerial and medical worlds

First, medical leaders perform activities in which they act as liaisons, that maneuver between different objectives to bridge the managerial and medical world (N = 12). Within this role, the medical leader acts as a coordinator [9], to create institutional linkages within and between organizations [38] and monitor and report information of interest back and forth between the managerial and medical worlds [11, 54]. This ‘linking-pin’ role is considered important for aligning the interests of both worlds [8]. Understanding the discourses of both worlds could enable the medical leader to bridge the gap [6, 31].

#### Influencing for multiple objectives

Second, many activities include methods to exert influence (N = 12) to serve objectives of the ‘self’ and the medical staff (N = 6), rather than only serving the organizational objectives. For instance, by influencing the expectations of peers and managers [30] or delegating and coordinating tasks [51], medical leaders can create their own preferable roles. Furthermore, decision making (N = 5), such as patient referrals, is a means of retaining professional autonomy and control over clinical issues [54]. Having a voice in strategy and decision making at ‘higher’ levels is important to guarantee that the medical staff interests are met [28, 43, 46, 51]. For example, financial activities may be a way to influence and control resource allocation [43]. Finally, medical leaders must influence and negotiate (N = 2) with, medical peers, non-medical managers, and stakeholders outside the hospital to acquiesce the changes that are necessary for organizational purposes. In the processes of negotiating or influencing, medical leaders must balance between clinical and organizational practices to safeguard both the quality *and* efficiency of care [2, 15, 53]. Some even argue that the effectiveness of these processes will increase if a physician acts as a leader instead of a manager who seeks to exercise authority over others [8, 15, 53].

#### Dealing with tensions

Third, to be able to balance between management and medicine, medical leaders must manage several tensions (N = 2). At the intrapersonal level, medical leaders must cope with their “multiple identities” as both physicians and managers. This balance is described as role-making activities (N = 6), such as sense making and identity work [8, 30, 32, 44, 51, 54]. At the organizational level, medical leaders must deal with tensions among individuals, competing departments, the medical and managerial world and external and internal organizational demands [54]. Notably, many studies show that medical leaders will always prioritize their clinical identity and activities over their managerial identity and activities [6, 34]. Finally, several studies reported that some physicians believed that certain activities belonged more to managers than to medical professionals, such as performance management [51], in which a subpar performance could be a threat to the physician’s clinical autonomy [54], and finance [43], allowing the medical leaders to be “free from day-to-day operational financial management” [ibid].

### Personal features related to medical leadership

At the personal level, features that refer to the characteristics of a medical leader were elicited (Table 3). These features include credibility (N = 22), skills (N = 21), knowledge (n = 14), attitude (N = 14), and experience in management (N = 12). We observed no distinction between formal and informal medical leadership roles. In the overview of these features presented in Table 3, the features that are distinctive for medical leaders compared to those that are distinctive of general leaders are marked with an asterisk.

**Table 3:** Personal features.

Authors (year of publication)	Credibility	Skills	Knowledge	Attitude	Experience in managerial work
Andersson 2015 [8]	Commitment to clinical work*	**-**	**-**	**-**	**-**
Barrable 1988 [34]	Medical excellence*, respected by peers, commitment to clinical work*	Conceptual, collaborative, empowering, lead by example, providing feedback, communication, staff management, resolve conflicts, administration, HR	Report writing, finance, IT, performance management, HR, logistics, health policy and law	Ethical and moral values, open-minded	Lack of experience in administration, need for training, concerns about performance
Betson & Pedroja 1989 [11]	Medical excellence*	**-**	**-**	**-**	Need for training
Buchanan et al. 1997 [12]	Medical excellence*, respected by peers	Vision, conceptual, teaching, time management, decision-making, self-regulation, collaborative, provide feedback, communication, listening, resolve conflicts, staff management, HR, negotiation, networking, delegation, administration, performance management, strategic, lead change, political, bridge medicine and management*, represent staff and specialty	Clinical*, leadership role, structure of the organization, health system, hospital market	Diplomatic, assertive, patience, personable, patient centered*, cooperative, motivated	Need for training
Dawson et al. 1995 [35]	Professional credibility	**-**	**-**	**-**	Lack of experience in similar jobs, need for training, concerns about performance
Dedman et al. 2011 [13]	Medical excellence*, respected, authority, trusted	Self-awareness, HR, collaborative, empowering, communication, performance management, strategic management, negotiation, political, administration, staff management	Clinical*, health system, public health	Diplomatic, motivated, patient centered*, honest, open-minded	Needs training
Dine et al. 2010 [36]	**-**	Vision, conceptual, time management, self-regulation, empowering, providing feedback, communication, team, resolve conflicts, performance management	Clinical	Enthusiasm for medicine*, integer, patient centered*, being visible, cooperative, quality driven, mission driven	**-**
Dwyer 2010 [28]	**-**	Writing, decision-making, self-regulation, collaborative, empowering, communication, staff management, resolve conflicts, administration, strategic, HR, quality improvement	Clinical*, health policy and law	**-**	**-**
Hallier & Forbes 2005 [37]	Commitment to clinical work*	**-**	**-**	**-**	Need for training
Holmboe et al. 2003 [38]	Medical excellence*, objectivity, quality improvement	Empowering, communication, resolve conflicts, networking, bridge management and medicine*	IT	Innovative, assertive, quality driven, mission driven	**-**
Hopkins et al. 2015 [39]	**-**	Conceptual, self-awareness, self-regulation, empowering, communication, team, resolve conflicts, negotiation, networking, administration, lead change	**-**	Self-confidence, assertive, persistent, adaptability, integer, open-minded, honest and open, empathetic, mission driven, result driven, forward thinking	**-**
Kindig & Lastirir-Quiros 1989 [40]	**-**	**-**	**-**	**-**	Need for training
Kippist & Fitzgerald 2009 [31]	**-**	Collaborative, performance management, political, bridge management and medicine*	Finance, performance management	**-**	Need for training
Kuhlmann et al. 2016 [41]	**-**	**-**	Clinical*	**-**	**-**
Leigh & Newman 1997 [42]	**-**	Communication			Concerns about financial ability
Llewellyn 2001 [43]	Medical excellence*, commitment to clinical work*	Administration	Clinical*, finance	**-**	Need for financial skills
Meier 2015 [2]	Medical excellence*, medical position*	**-**	**-**	**-**	**-**
Mo 2008 [44]	Commitment to clinical work*	**-**	Clinical*	**-**	**-**
Ong 1998 [32]	**-**	Strategic	**-**	**-**	Lack of experience in similar job, need for training
Palmer at al. 2009 [45]	**-**	Vision, conceptual, self-awareness, collaborative, empowering, strategic, lead change	**-**	Self-confidence, intellect, integer, cooperative, result driven	**-**
Pepermans et al. 2001 [47]	**-**	**-**	**-**	**-**	**-**
Quinn & Perelli 2016 [46]	Medical excellence*	**-**	**-**	**-**	**-**
Robinson et al. 2013 [48]	Medical excellence*, commitment to clinical work*, trusted	Collaborative, empowering	**-**	Personable, integer, result driven, forward thinking, cooperative	Need for training
Rotar et al. 2016 [49]	**-**	**-**	**-**	**-**	**-**
Spehar et al. 2015 [50]	Medical excellence*, commitment to clinical work*	Listening	**-**	Visible	**-**
Spyridonidis et al. 2015 [51]	Professional autonomy	**-**	**-**	**-**	**-**
Taylor et al. 2008 [52]	Medical excellence*	Vision, self-awareness, self-regulation, communication, listening	Clinical*, finance, IT, structure of the organization	Motivated, empathetic	**-**
Thorne 1997a [53]	Committed to clinical work*	**-**	**-**	**-**	Lack of experience in similar jobs, concerns about performance
Thorne 1997b [54]	Medical excellence*, collegial disposition, ethical and moral values	Empowering, communication, resolve conflicts, negotiation, networking, run meetings	Clinical*, structure of the organization, strategy, marketing	Motivated, contract focused	**-**
Vinot 2014 [9]	Medical excellence*, ability to bridge management and medical worlds*	Team, staff management, negotiation, bridge management and medicine*, networking	**-**	**-**	**-**
Willcocks 1995 [30]	Medical excellence*, respected by peers	Time management, collaborative, communication, resolve conflicts, administration, strategic, marketing	Clinical*, finance, leadership role, structure of the organization, health system and sector	Motivated, customer focused	Lack of experience, concerns about financial ability
Williams 2001 [10]	**-**	Conceptual, writing, time management, decision-making, vision, empowering, lead by example, build trust, communication, team, listening, resolve conflicts, negotiation, networking, HR, lead change, administration, strategic, run meetings, risk management, contracting	Clinical*, finance, IT, performance management, strategy, quality assurance, marketing, health system, policy and law, hospital market	Assertive	**-**
Williams & Ewell 1996 [55]	Medical excellence*, respected by peers, experience in committees	Vision, decision-making, dealing with uncertainty, collaborative, empowering, communication, listening, resolve conflicts, negotiate, administration, lead change, political, run meetings	Strategy, marketing	Assertive, objective, stress-resistant, innovative, intellect, creative, ethical and moral values, patient centered*	**-**
Witman et al. 200 [6]	Medical excellence*, scientific disposition, respected by peers, collegial disposition, trusted	Negotiation	**-**	**-**	**-**

* Features indicated with an asterisk indicate the unique features of medical leadership in contrast to those of general leadership

First, 21 studies showed the importance of *credibility* among medical peers in executing both clinical and managerial careers. Credibility is an important source of legitimacy, influence and recognition, which are required to ‘get things done’. For example, the reputation of clinical excellence “has put clinical directors in a relatively strong position vis-à-vis management” [43]. Moreover, credibility and maintaining a clinical identity are important for medical leaders’ clinical careers, as many of these physicians hope to return to full-time clinical work ‘someday’. Furthermore, the retention of a professional focus and identification was considered important in preventing isolation from medical peers not only because they do not want to be considered managers but also to convince colleagues that they did not choose the management track because they failed in their clinical careers [44]. Credibility could be obtained in several ways. The most important sources of credibility are medical excellence (N = 16), commitment to clinical work (N = 4), and thereby thus “showing where their real allegiances lay” [43], respect by peers (N = 6), trust by peers (N = 3), and a collegial disposition (N = 2).

Second, 21 studies reported on the required *skills* for medical leaders. The most cited skills include communication (N = 12), empowering others (N = 11), resolving conflicts (N = 10), administrative skills (N = 9), collaborative skills (N = 9) and negotiating (N = 8), followed by strategic skills (N = 7), leading change (N = 6), team skills (N = 6), the ability to carry out a vision (N = 6), networking (N = 6), and conceptual skills (N = 6). The arguments supporting the importance of these items differed. Some authors mentioned that these skills were required for conducting general management or leadership work [52]. Furthermore, it was argued that these skills were necessary to negotiate for or represent the interests of the entire organization [13, 35, 52, 54]. Other authors explicitly emphasized that these skills are necessary for balancing between medicine and management [9, 12, 28, 31, 38] or negotiating for and representing the interests of the medical staff [6, 9, 43]. However, other authors did not explain why these specific items must be acquired or possessed by a medical leader [10, 30, 36, 55].

Third, 14 studies reported the importance of different areas of *knowledge*. The most cited area of knowledge was clinical knowledge (N = 9). Some arguments were rather straightforward, such as that clinical knowledge is crucial for making informed decisions at the departmental level [44] or convenient for attracting additional contracts [43]. Other arguments appeared to be related to retaining power and control rather than clinical issues. In their study investigating clinical directors, Dedman et al. [13], showed physicians argued that clinical knowledge was necessary to ensure that “decisions are based on clinical evidence”, and Llewellyn [43] that physicians that argued that, in specific areas, clinical knowledge was necessary because “managers cannot escape from the ultimate authority of doctors” [ibid]. Other areas of required knowledge that were mentioned more than twice included finance (N = 6), IT (N = 4), organizational structures (N = 4), the health system (N = 4), health policy and law (N = 3), marketing (N = 3) and performance management (N = 3).

Fourth, 14 studies described *attitudes* (or *traits*), i.e., the innate personal qualities and characteristics that medical leaders should possess. The most cited traits were motivation (N = 5), assertiveness (N = 5), cooperativeness (being a team player) (N = 4), patient centered (N = 4), integrity (N = 4), mission driven (N = 3) and result driven (N = 3), followed by diplomatic (N = 2), personable (N = 2), honest and open (N = 2), visible (N = 2), quality driven (N = 2), innovative (N = 2), self-confident (N = 2), empathetic (N = 2), forward thinking (N = 2), and intellect (N = 2). Taylor et al. [52] questioned whether these attitudes can be learned or whether they are innate. They argue that having a mission is an innate trait, whereas areas of knowledge (such as finance) or skills (such as networking) can be learned.

Fifth, 12 studies provided evidence that physicians in formal managerial roles felt that they lacked *experience* in the ‘unknown’ field of management N = 5). In some cases, this lack of experience led to feelings of insecurity regarding the quality of their performance as managers (N = 2) or concerns regarding their financial skills (N = 2). Moreover, these physicians mentioned the difficulties in evaluating their performance because often, no formal feedback was provided by others. This difficulties could be serious issues for physicians because the physicians felt that they must always avoid public [53] due to the importance of status and credibility. To overcome these issues, many studies reported the possibility of following learning programs to obtain specific management or leadership skills and knowledge (N = 10). However, the argument to undergo training appeared to have additional objectives as follows: both as a tactic to ensure that the physicians could not get ‘overruled’ by management, i.e., through management jargon, and a strategy to have influence over financial or organizational issues such as resource allocation [43].

### Context-specific features related to medical leadership

The following section presents the features that are related to the specific hospital-context in which a medical leader operates that may be perceived as either barriers or a facilitators (Table 4). This category includes the factors of competing logics (N = 16), time (N = 14), role ambiguity (N = 13), and support (N = 11).

**Table 4:** Context-specific features.

**Authors (year of publication)**	**Competing logics**	**Lack of time**	**Role ambiguity**	**Lack of support**
Andersson (2015) [8]	Identity struggles	-	-	-
Barrable (1988) [34]	-	More time needed for leadership role	Lack of clarity about job content	-
Betson & Pedroja (1989) [11]	-	-	-	-
Buchanan et al. (1997) [12]	Management versus clinical work, different objectives	Threat to clinical work, more time needed for leadership role, work overload	Lack of clarity about job content	Lack of support
Dawson et al. (1995) [35]	Management versus clinical work	Threat to clinical work, work overload	-	Importance of support of clinical colleagues and executives, no financial reimbursement
Dedman et al. (2011) [13]	Different objectives	-	Lack of clarity about job content	-
Dine et al. (2010) [36]	-	-	-	-
Dwyer (2010) [28]	-	-	-	-
Hallier & Forbes (2005) [37]	Management versus clinical work, distrust	-	Lack of clarity about job content	Lack of support (of executives and clinical colleagues), no formal responsibility, no financial reimbursement
Holmboe et al. (2003) [38]	-	-	-	-
Hopkins et al. (2015) [39]	-	-	-	-
Kindig & Lastirir-Quiros (1989) [40]	-	-	-	-
**Authors (year of publication)**	**Competing logics**	**Lack of time**	**Role ambiguity**	**Lack of support**
Kippist & Fitzgerald (2009) [31]	-	Management versus clinical work, more time needed for leadership role, work overload	Lack of clarity about job content, lack of job description, opportunity for role making	Lack of formal responsibility
Kuhlman et al. 2016 [41]	Identity struggles	-	Lack of clarity about job content	Lack of organizational support, lack of acceptance within the medical field, lack of formal responsibility
Leigh & Newman (1997) [42]	Tensions	Time consuming	-	No support (of secretaries and assistants), no financial reimbursement
Llewellyn (2001) [43]	Distrust, different objectives	Threat to clinical work and credibility	-	-
Meier (2015) [2]	-	-	-	-
Mo (2008) [44]	Distrust, different objectives	Threat to clinical work	-	-
Ong (1998) [32]	Tensions, different objectives	More time needed for leadership role	No role models, lack of clarity about job content, no role recognition, opportunity for role making	No support (by executives and clinical colleagues), isolation
Palmer et al. (2009) [45]	-	-	-	-
Pepermans et al. (2001) [47]	-	-	-	-
Quinn &Perelli 2016 [46]	Identity struggles, tensions	Time consuming, threat to clinical work and credibility	Lack of clarity about job content	No financial reimbursement
Robinson et al. (2013) [48]	-	Lack of time	Lack of job description	-
**Authors (year of publication)**	**Competing logics**	**Lack of time**	**Role ambiguity**	**Lack of support**
Rotar et al. 2016 [49]	-	-	-	-
Spehar et al. (2015) [50]	Identity struggles, management versus clinical work	More time needed for leadership role, work overload	-	-
Spyridonidis et al. (2015) [51]	-	-	Opportunity for role making	Support as interference
Taylor et al. (2008) [52]	-	-	-	-
Thorne (1997a) [53]	Management versus clinical work	-	No role models	Trust of colleagues needed
Thorne (1997b) [54]	Identity struggles, tensions, distrust	Work overload	Lack of job description, lack of clarity about job content, opportunity for role making	Lack of support (of executives and clinical colleagues)
Vinot (2014) [9]	-	-	-	-
Willcocks (1995) [30]	Identity struggles, management versus clinical work	Threat to clinical work	Lack of clarity about job content	-
Williams (2001) [10]	-	-	-	-
Williams & Ewell (1996) [55]	-		-	-
Witman et al. (2010) [6]	Different objectives	Management versus clinical work, threat to clinical work and credibility	-	-

First, many studies reported the issue of *competing logics* (N = 16), often leading physicians to feel ‘stuck’ and having to choose between two worlds. While performing their hybrid role, medical managers encounter several dichotomies, such as quality of care *versus* efficiency [12], working autonomously *versus* being a subordinate [8] and engaging in clinical work *versus* managerial work [6]. Notably, most experienced meaning, satisfaction and legitimacy in clinical work. For example, physicians will never identify primarily as managers [32, 43, 46, 50]. To overcome these dichotomies, only four studies noted the importance of finding a common ground between management and medicine [32, 35, 43, 54].

Second, many studies emphasized *lack of time* (N = 14) as a significant burden. Time issues were mostly about dividing time between clinical and managerial work [6], as many physicians only performed managerial activities part-time. Moreover, regarding the importance of credibility, the physicians did not want to spend too much time on managerial work because they feared losing credibility among their medical peers [43] and it is not considered a career step [31]. Consequently, many physicians experienced that managerial work came ‘on top’ of or ‘alongside’ their clinical work, resulting in overtime work, stress, exhaustion and dissatisfaction.

Third, several studies described *role ambiguity* (N = 13) as an influential factor in performing medical leadership roles. Medical leaders perceived this role ambiguity as either negative or positive. The lack of a well-defined role description, such as a description including activities and formal responsibilities, was experienced as a barrier that resulted in stress [54], concerns [30] and frustration [13]. Moreover, the new role led to unwanted tasks, such as managing medical colleagues—who may have been previously ignored—and addressing conflicts and resistance. Furthermore, the responsibility for the performance of medical peers could pose a threat to their clinical autonomy [54]. These tensions were a source of frustration and often led to stress and uncertainty. However, for some, the lack of a role definition provided opportunities as the role became “more fluid and open for interpretation” [51]. Notably, managers often describe the role as a way to ‘control physicians’, while physicians describe the role as ‘protecting’ physicians from management [54].

Fourth, many authors mentioned the importance of *support* (N = 11) in becoming an effective medical manager. The importance of support and trust is two-fold. On the one hand, medical managers need ‘backing’ from their medical colleagues, as they (the medical managers) must ‘protect’ them (the medical staff) from the management world. On the other hand, physicians must gain support and trust from the management world to obtain authority and responsibility and prevent exclusion from key decisions and strategy. In conclusion, support and trust form an important component of a medical manager’s power base [35] and are needed not only to become effective but also to prevent stress and working in isolation [41]. Paradoxical, a few authors [51, 54] mentioned that in some cases, physicians explicitly did not want support because sharing their problems could lead to public failure and the loss of credibility and status.

## Discussion

In this study, we presented a systematic overview of the literature on medical leadership in hospital settings to improve the empirical knowledge and conceptualization of the subject. Accordingly, we analyzed the included records in terms of (1) definitions, (2) activities and roles, (3), skills, and (4) influential factors. We provide a comprehensive framework, including an overview of the features that are related to medical leadership at the personal, context-specific and role-related levels.

Based on our findings, we can distinguish between two types of medical leadership conceptualizations. Type 1 medical leadership includes physicians working in formal leadership roles who are mostly defined as medical managers who work at either the management or executive level at a hospital in addition to or instead of their clinical practice. Type 2 medical leadership includes physicians working in informal leadership roles at the clinical level, i.e., these physicians act as leaders within their daily clinical practice.

Regardless of the type of role a medical leader performs, this role appears to be two-fold. On the one hand, this role entails broad range of general management and leadership activities on the other hand, this role entails activities to balance between the management and medical worlds. These activities must be performed not only to achieve and represent organizational objectives but also to negotiate for and represent the interests of the medical staff. To pursue these interests, the included studies revealed a multiplicity of general management and leadership skills, knowledge and attitudes, specific skills, and knowledge and attitudes specific to balance between management and medicine.

Although most elicited activities, skills, knowledge and attitudes were not different from those identified by well-established researchers in the field of general leadership [57]–and could thus be applicable to leaders with other backgrounds–our findings showed that *medical* leadership differs from general leadership. An important facet of a medical leadership role includes balancing between management and medicine, which forces medical leaders to constantly maneuver between clinical and organizational objectives to safeguard both the quality *and* efficiency of care. A medical background appeared to be crucial for conducting these boundary-spanning roles. Our study revealed the importance of two personal features–perceived as either barriers or facilitators–that appeared to specifically apply to a leader with a *medical background*, namely credibility and clinical knowledge. Credibility, in terms of medical excellence, a medical position within a hospital, an ability to bridge the management and medicine worlds and a commitment to clinical work is considered important for effectively performing a medical leader role. Clinical knowledge is considered important and distinctive for an effective medical leader in comparison to a general leader without a clinical background because, clinical knowledge cannot be easily acquired by a leader without a medical background, whereas in other fields, knowledge may be more easily acquired [43]. Considering the specific hospital setting in which a medical leader operates, we found four features that are related to the specific context in which a medical leader performs her/his role, namely, the existence of competing logics in hospitals, role ambiguity, and (the lack of) support and time.

Although our systematic review provides conceptual clarity, a few issues remain unaddressed. The definitional issues, the number and diversity of the elicited activities, the personal and context-specific features, the role ambiguity and the lack of time, support and experience show the lack of standardization and institutionalization of medical leadership in practice and the lack of conceptual clarity in the literature. Moreover, we did not observe a clear distinction between formal and informal medical leadership roles or among the levels at which a medical leader could be active (i.e., executive, management or clinical) in terms of activities and the personal- and context-specific features. This finding raises a few fundamental questions. First, to what extent do physicians need to master *all* the elicited items and, more specifically, be involved in managerial domains? Second, what are the potential consequences on professional work and the boundaries of the professional domain? In our findings, we observed a distinction between soft skills, such as communication and collaboration, and more technical skills, such as administration and finance, which raises questions regarding whether medical leadership entails a *shift* or a *reallocation* of tasks. Subsequently, this finding leads to the question of the extent to which medical leaders should be accountable for the performance of these ‘new’ activities. Third, what are the potential consequences for medical training? In practice, competency models for physicians have recently changed ‘management’ to ‘leadership’ as an important component of current professional work [5]. However, an increased understanding of the exact content of this concept is necessary to improve medical training.

Furthermore, our findings show the difficulty of ‘simply’ involving physicians in leadership or managerial positions to improve medical governance and overcome the divide between the managerial and medical world. Medical leaders often create their own roles and subsequently use their hybrid role to exert an influence on organizational issues and serve not only the organizational objectives but also their own or the medical staff’s objectives. In the literature, this is also known as *restratification*, which was proposed by Freidson [58], who described this phenomenon as a means of maintaining professional authority in healthcare [59]. Restratification questions whether the competing logics could disappear by incorporating organizational work into the physicians’ daily practices.

Regarding future research, we have several empirical and theoretical recommendations. To further increase conceptual clarity, more in-depth studies implementing stronger research designs are needed. For example, in all quantitative studies, a survey was used to rank predefined lists of either skills or activities. Although this offers insights into the participants’ views regarding the importance of the particular activities or skills, it does not show the *relative* importance of these items for effective medical leadership. Investigating which items are of relatively higher importance for effective medical leadership could further increase the conceptual clarity because the outcomes of this review and the multiplicity of the activities and personal–and context-specific features, question whether medical leaders need to master *all specific* items. Furthermore, future studies can use the framework of medical leadership in hospital settings (Fig 2) in which we point out possible directions for future research. Future studies can elaborate on the framework to investigate the extent to which the features relate to each other and the effectiveness of these features in relation to outcomes such as quality, efficiency or safety of care in hospital settings. In doing so, future studies can develop an evidence-based framework for medical leaders in hospital settings which can be used for further research or leadership development purposes. Finally, and arguably a limitation of this review, it is currently unclear whether the elicited activities and the personal and context-specific features are of equal importance for different medical specialties and for physicians working outside a hospital setting, such as general practitioners.

Another methodological suggestion concerns the predominant literature focus on the formal roles of medical leadership. Therefore, a better understanding of the informal roles of medical leadership is necessary to increase our knowledge of the changing nature of the medical profession and *how* physicians can incorporate organizational work into their daily practices, thereby acting as ‘leaders’. Ethnography is a fruitful method for obtaining insight into daily leadership practices in organizations (in this case, hospitals). Sutherland [60] argues that ethnography remains an underrepresented method of studying leadership, although it offers important knowledge regarding the subtle processes of leadership, such as meaning making, argumentation and negotiation. The use of ethnography could improve our understanding of *how* medical leadership originates in practice and is therefore a welcomed addition to our current knowledge regarding medical leadership.

Finally, we have two theoretical suggestions for future studies. First, more knowledge is required regarding the *identity and institutional work* performed by medical leaders perform and their consequences, because this review revealed that many medical leaders struggle with their new identity and the fact that the new role they must perform is not well formalized and institutionalized. The use of institutional and identity theories [61] to investigate these issues may be helpful. Second, it would be fruitful to study how medical leaders perform *boundary work* [62] and how this affects the quality and efficiency of care because our study showed that medical leaders are often ‘caught up’ in boundary work, and they must balance between and, more importantly, link managerial and medical logics. As Ong [32] suggests, it is important to search for common ground as shared objectives, and maybe even more fundamentally, mutual understanding may help to eliminate dichotomization in hospitals.

## Supporting information

S1 Appendix Example of the full electronic search strategy for all databases. (PDF)

S1 Table PRISMA checklist. (PDF)

S1 File First title and abstract screening 2016. (XLSX)

S2 File First full text screening 2016. (XLSX)

S3 File Updated title and abstract screening 2017. (XLSX)

S4 File Updated full text screening 2017. (XLSX)
