# Supplementary material for: Medical leaders or masters?—A systematic review of medical leadership in hospital settings
Source: PLoS One. 2017 Sep 14;12(9):e0184522. doi: 10.1371/journal.pone.0184522 (PMC5598981; doi:10.1371/journal.pone.0184522)
Supplement: S1 Appendix — (PDF) [file pone.0184522.s001.pdf]

Embase.com	5620
Medline ovid	4022
Web of science	3162
Cochrane	64
Cinahl (ebscohost)	2266
ABIinform (ProQuest)	734
Google scholar	200
**Total**	**16065**

Embase.com

((((medical OR clinical OR physician* OR clinician* OR doctor*) NEXT/1 (director* OR leader* OR manager* OR executive* OR engage* OR ceo OR ceos)) OR ((physician* OR clinician* OR 'healthcare professional' OR 'healthcare professionals') NEAR/6 (leadership OR management OR 'in charge' OR champion) NEXT/1 (role* OR position*))):ab,ti AND ('hospital management'/de OR 'hospital personnel management'/de OR 'health care organization'/de OR 'organization and management'/de OR 'administrative personnel'/de OR 'manager'/de OR 'health care quality'/de OR 'health care management'/de OR 'health personnel attitude'/de OR 'professional competence'/de OR 'hospital administrator'/de OR skill/de OR (((hospital OR 'health care' OR medical) NEAR/6 (management* OR organization* OR qualit* OR system* OR administrat* OR director*)) OR (organization NEAR/3 management) OR (administrative NEAR/3 (personnel* OR management*)) OR competenc* OR ((personal) NEXT/1 skill*) OR (leadership NEAR/6 (skill* OR Prerequisite* OR Requirement* OR Context* OR Condition* OR Precondition* OR Organizational OR Barrier* OR Arrangement* OR Tool*)) OR (Chief* NEAR/3 (staff* OR executive* OR officer*)) OR ceo OR ceos OR (board NEXT/1 member*)):ab,ti) OR ((medical OR clinical) NEXT/1 leadership*):ab,ti) NOT ([Conference Abstract]/lim OR [Letter]/lim OR [Note]/lim OR [Editorial]/lim) AND [english]/lim

Medline ovid

((((medical OR clinical OR physician* OR clinician* OR doctor*) ADJ (director* OR leader* OR manager* OR executive* OR engage* OR ceo OR ceos)) OR ((physician* OR clinician* OR "healthcare professional" OR "healthcare professionals") ADJ6 (leadership OR management OR "in charge" OR champion) ADJ (role* OR position*))).ab,ti. AND ("Hospital Administration"/ OR "Organization and Administration"/ OR "Administrative Personnel"/ OR "Quality of Health Care"/ OR "Attitude of Health Personnel"/ OR "Professional Competence"/ OR "Hospital Administrators"/ OR (((hospital OR "health care" OR medical) ADJ6 (management* OR organization* OR qualit* OR system* OR administrat* OR director*)) OR (organization ADJ3 management) OR (administrative ADJ3 (personnel* OR management*)) OR competenc* OR ((personal) ADJ skill*) OR (leadership ADJ6 (skill* OR Prerequisite* OR Requirement* OR Context* OR Condition* OR Precondition* OR Organizational OR Barrier* OR Arrangement* OR

Tool*)) OR (Chief* ADJ3 (staff* OR executive* OR officer*)) OR ceo OR ceos OR (board ADJ member*)).ab,ti.) OR ((medical OR clinical) ADJ leadership*).ab,ti.) NOT (letter OR news OR comment OR editorial OR congresses OR abstracts).pt. AND english.la.

Cochrane

((((medical OR clinical OR physician* OR clinician* OR doctor*) NEXT/1 (director* OR leader* OR manager* OR executive* OR engage* OR ceo OR ceos)) OR ((physician* OR clinician* OR 'healthcare professional' OR 'healthcare professionals') NEAR/6 (leadership OR management OR 'in charge' OR champion) NEXT/1 (role* OR position*))):ab,ti AND ((((hospital OR 'health care' OR medical) NEAR/6 (management* OR organization* OR qualit* OR system* OR administrat* OR director*)) OR (organization NEAR/3 management) OR (administrative NEAR/3 (personnel* OR management*)) OR competenc* OR ((personal) NEXT/1 skill*) OR (leadership NEAR/6 (skill* OR Prerequisite* OR Requirement* OR Context* OR Condition* OR Precondition* OR Organizational OR Barrier* OR Arrangement* OR Tool*)) OR (Chief* NEAR/3 (staff* OR executive* OR officer*)) OR ceo OR ceos OR (board NEXT/1 member*)):ab,ti) OR ((medical OR clinical) NEXT/1 leadership*):ab,ti)

Web of science

TS=(((((medical OR clinical OR physician* OR clinician* OR doctor*) NEAR/1 (director* OR leader* OR manager* OR executive* OR engage* OR ceo OR ceos)) OR ((physician* OR clinician* OR "healthcare professional" OR "healthcare professionals") NEAR/5 (leadership OR management OR "in charge" OR champion) NEAR/1 (role* OR position*))) AND ((((hospital OR "health care" OR medical) NEAR/5 (management* OR organization* OR qualit* OR system* OR administrat* OR director*)) OR (organization NEAR/2 management) OR (administrative NEAR/2 (personnel* OR management*)) OR competenc* OR ((personal) NEAR/1 skill*) OR (leadership NEAR/5 (skill* OR Prerequisite* OR Requirement* OR Context* OR Condition* OR Precondition* OR Organizational OR Barrier* OR Arrangement* OR Tool*)) OR (Chief* NEAR/2 (staff* OR executive* OR officer*)) OR ceo OR ceos OR (board NEAR/1 member*))) OR ((medical OR clinical) NEAR/1 leadership*))) AND DT=(article)

Cinahl (ebscohost)

((((medical OR clinical OR physician* OR clinician* OR doctor*) W1 (director* OR leader* OR manager* OR executive* OR engage* OR ceo OR ceos)) OR ((physician* OR clinician* OR "healthcare professional" OR "healthcare professionals") N5 (leadership OR management OR "in charge" OR champion) W1 (role* OR position*))) AND (MH "Health Facility Administration" OR "Management" OR MH "Administrative Personnel" OR MH "Quality of Health Care" OR MH "Attitude of Health Personnel" OR MH "Professional Competence" OR MH "Health Facility Administrators" OR (((hospital OR "health care" OR medical) N5 (management* OR organization* OR qualit* OR system* OR administrat* OR director*)) OR (organization N2 management) OR (administrative N2 (personnel* OR management*)) OR competenc* OR ((personal) W1 skill*) OR (leadership N5 (skill* OR Prerequisite* OR Requirement* OR Context* OR Condition* OR Precondition* OR Organizational OR Barrier* OR Arrangement* OR Tool*)) OR (Chief* N2 (staff* OR executive* OR officer*)) OR ceo OR ceos OR (board N1 member*))) OR ((medical OR

clinical) W1 leadership*)) NOT PT (letter OR news OR comment OR editorial OR congresses OR abstracts) AND LA (english)

ABIinform (ProQuest) - Scholarly journals

AB,TI(((((medical OR clinical OR physician* OR clinician* OR doctor*) P/1 (director* OR leader* OR manager* OR executive* OR engage* OR ceo OR ceos)) OR ((physician* OR clinician* OR "healthcare professional" OR "healthcare professionals") N/5 (leadership OR management OR "in charge" OR champion) P/1 (role* OR position*))) AND ((((hospital OR "health care" OR medical) N/5 (management* OR organization* OR qualit* OR system* OR administrat* OR director*)) OR (organization N/2 management) OR (administrative N/2 (personnel* OR management*)) OR competenc* OR ((personal) P/1 skill*) OR (leadership N/5 (skill* OR Prerequisite* OR Requirement* OR Context* OR Condition* OR Precondition* OR Organizational OR Barrier* OR Arrangement* OR Tool*)) OR (Chief* N/2 (staff* OR executive* OR officer*)) OR ceo OR ceos OR (board P/1 member*))) OR ((medical OR clinical) P/1 leadership*))) NOT PT (letter OR news OR comment OR editorial OR congresses OR abstracts) AND LA (english)

Google scholar

"medical|clinical leadership"
